# Deletion of 2 amino acids in *IHH* in a Japanese family with brachydactyly type A1

**DOI:** 10.1186/s12920-021-01042-6

**Published:** 2021-07-27

**Authors:** Nozomu Ozaki, Hiroko Okuda, Hatasu Kobayashi, Kouji H. Harada, Sumiko Inoue, Shohab Youssefian, Akio Koizumi

**Affiliations:** 1 Department of Pediatrics, Kadono-Sanjo Children’s Clinic, Kyoto, Japan; 2 Department of Pain Pharmacogenetics, Kyoto University Graduate School of Medicine, Kyoto, Japan; 3 Environmental and Molecular Medicine, Mie University Graduate School of Medicine, Tsu, Japan; 4 Department of Health and Environmental Sciences, Kyoto University Graduate School of Medicine, Kyoto, Japan; 5 Department of Molecular Biosciences, Kyoto University Graduate School of Medicine, Kyoto, Japan; 6 Institute of Public Health and Welfare, Kyoto-Hokenkai, Kyoto, Japan

**Keywords:** Brachydactyly type A1, *IHH* gene, Short stature

## Abstract

**Background:**

Brachydactyly type A1 (BDA1) is an autosomal dominant disorder characterized by uniform shortening of the middle phalanges in all digits. It is associated with variants in the Indian Hedgehog (*IHH*) gene, which plays a key role in endochondral ossification. To date, heterozygous pathogenic *IHH* variants involving several codons, which are restricted to a specific region of the N-terminal active fragment of *IHH*, have been reported. The purpose of this study was to identify the pathogenic variant in a Japanese family with BDA1 and to evaluate its pathogenesis with regard to previous reports.

**Methods:**

The proband, a 9-year-old boy, his siblings, and his father had shortened digits and a short stature of variable severity. Based on physical examinations, radiographic findings and family history, they were diagnosed with BDA1. This family is the first case of an isolated malformation in Japan. Sanger sequencing of *IHH* was performed on these individuals and on the proband’s unaffected mother. The significance of the variants was assessed using three-dimensional analysis methods.

**Results:**

Sanger sequencing showed a novel *IHH* heterozygous variant, NM_002181.4:c.544_549delTCAAAG(p.Ser182Lys183del) [NC_000002.12:g.219057461_219057466del].. These two residues are located outside the cluster region considered a hotspot of pathogenic variants. Three-dimensional modelling showed that S182 and K183 are located on the same surface as other residues associated with BDA1. Analysis of residue interactions across the interface between IHH and its interacting receptor protein revealed the presence of hydrogen bonds between them.

**Conclusions:**

We report a novel variant, NM_002181.4:c.544_549delTCAAAG (p.Ser182Lys183del) [NC_000002.12:g.219057461_219057466del] in a Japanese family with BDA1. Indeed, neither variations in codons 182 or 183 nor with such two-amino-acid deletions in IHH have been reported previously. Although these two residues are located outside the cluster region considered a hotspot of pathogenic variants, we speculate that this variant causes BDA1 through impaired interactions between IHH and target receptor proteins in the same manner as other pathogenic variants located in the cluster region. This report expands the genetic spectrum of BDA1.

## Background

Brachydactyly A1 (BDA1; MIM 112500) is inherited as an autosomal dominant disorder and is characterized by shortening of the middle phalanges of all the digits. The middle phalanges are either rudimentary or fused with the terminal phalanges. Approximately half of BDA1 cases are due to variants in the Indian Hedgehog (*IHH*) gene [1]. As a central signalling molecule mediating skeletal development, IHH plays an important role in modulating skeletal condensation, the growth and differentiation of chondrocytes, joint development and bone formation [2]. To date, more than 10 pathogenic variants in *IHH* have been identified in individuals with BDA1, and these variants cluster in the central region of the N-terminal active fragment [3]. Previous research has demonstrated that these residues constitute an interface that binds receptor proteins in a calcium-dependent manner, and the disruption of these calcium-dependent interactions by the variants causes BDA1. This is interpreted as a common mechanism of the BDA1-causing variants [4]. Most *IHH* variants have been characterized as missense [1, 3, 5–12], except for one insertion [13] and one deletion [14].

Here, we report a five-generation Japanese family with BDA1. Although the novel two-amino-acid deletions of codons 182 and 183 revealed by Sanger sequencing were located outside the cluster regions of pathogenic variants reported previously, which are considered to constitute an interface that binds receptor proteins [3], a three-dimensional analysis of IHH demonstrated that Ser182 and Lys183 are placed on the same surface as other previously reported mutations. We studied the pathogenesis of this variant through genetic and structural analyses.

## Methods

### Subjects

**Fig. 1:**
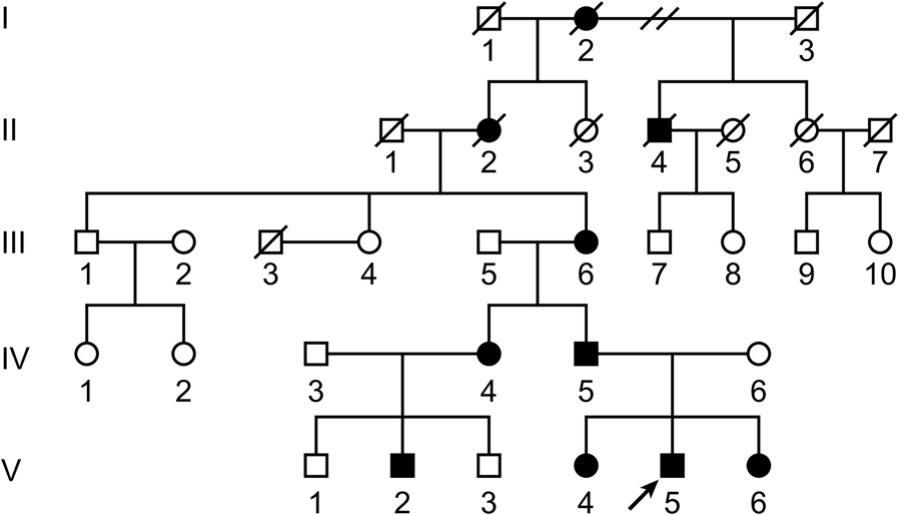
Pedigree of a five-generation family with brachydactyly type A1 (BDA1). Filled symbols represent affected individuals; open symbols represent unaffected individuals; squares represent males, and circles represent females. Diagonal lines indicate deceased individuals. The proband is indicated by an arrow

**Fig. 2:**
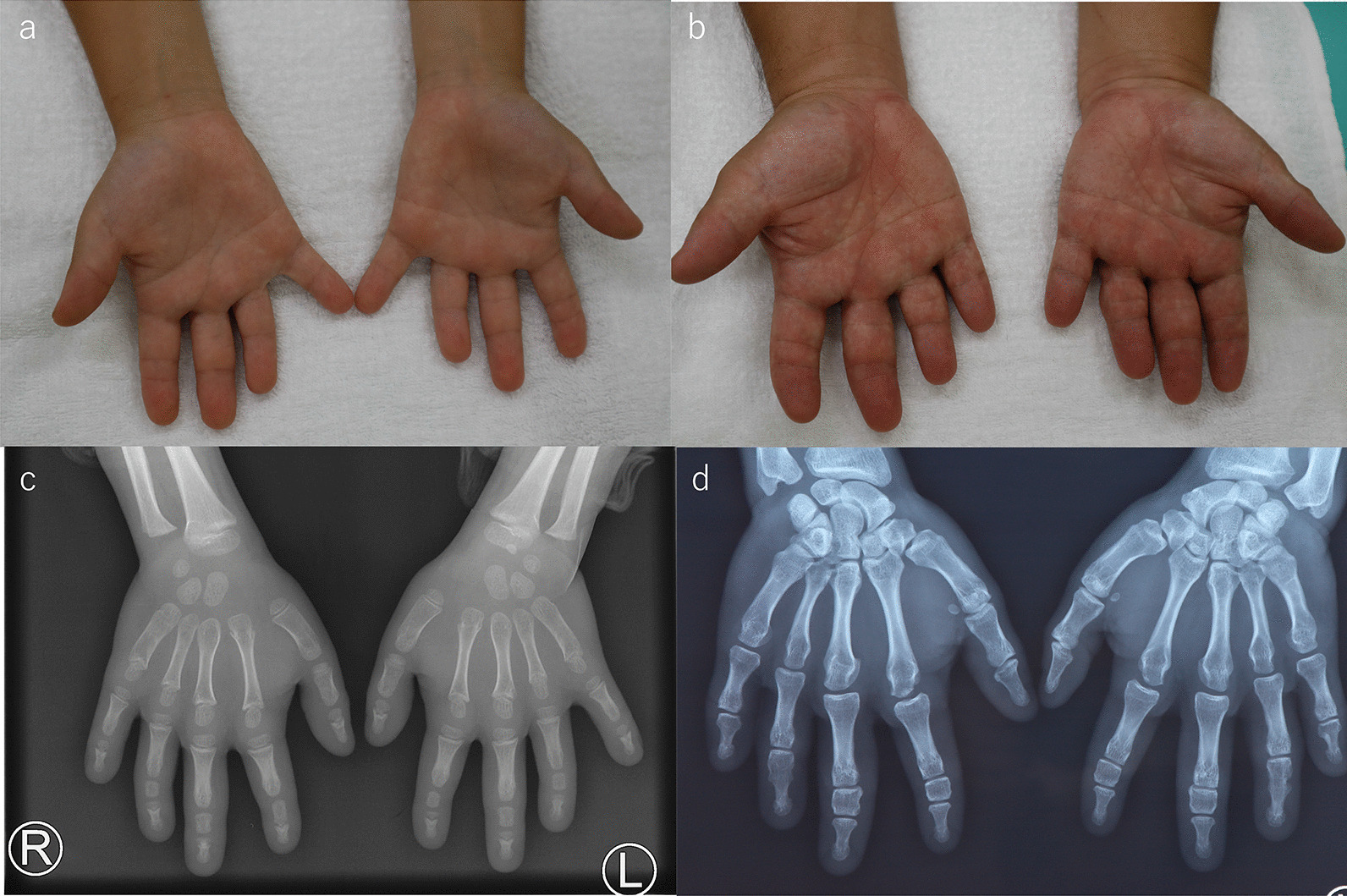
**a**–**d** The appearance and radiographic findings of the proband and his father with BDA1. **a**, **b** Proband and his father, respectively, showing shortening of all digits, missing interphalangeal creases in the third to fifth digits and mild clinodactyly of the fourth digit**. c **Proband showing shortening of the middle phalanges of the second to fifth digits and fusion of the middle and terminal phalanges of the fifth digit. **d** Father showing severe shortening of the middle phalanges of the second and third digits, and fusion of the middle to the terminal phalanges of the fourth and fifth digits

**Fig. 3:**
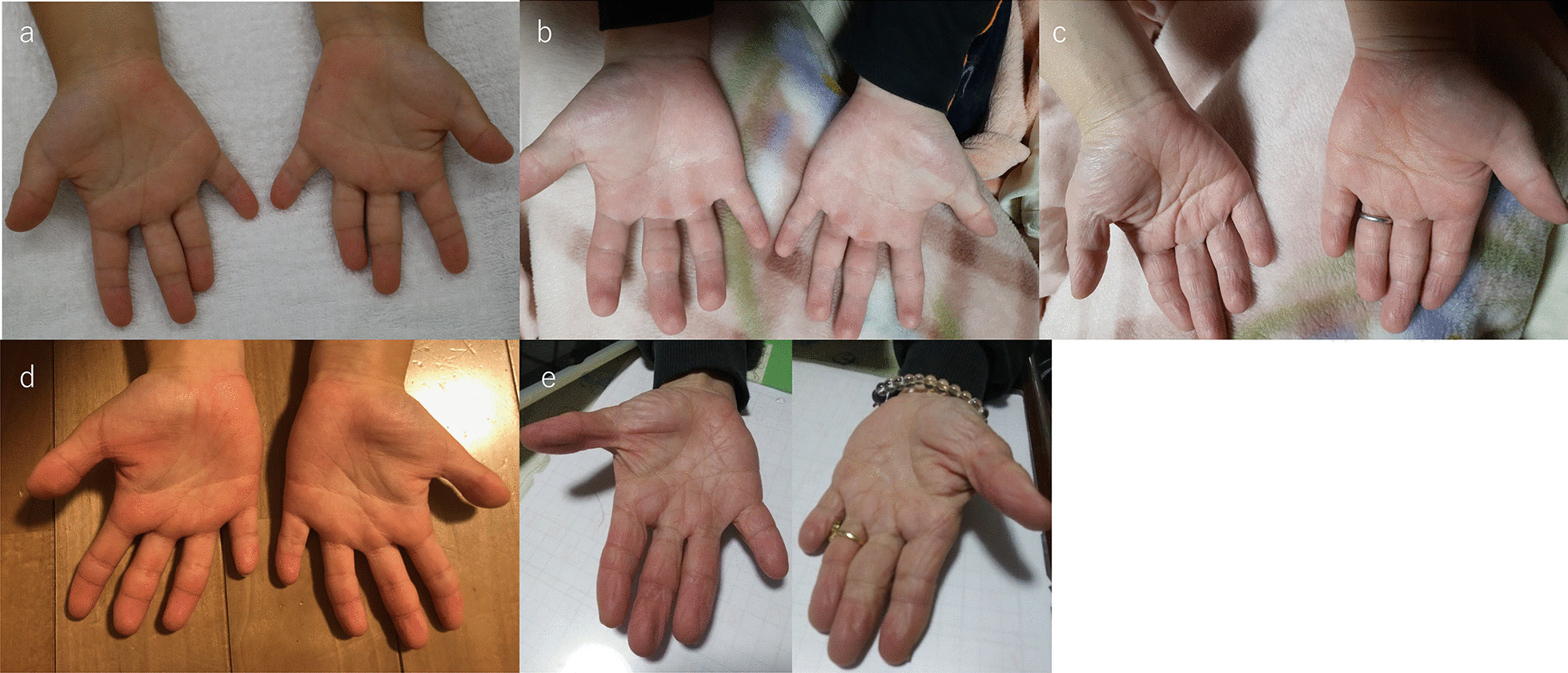
**a**–**e** Features of other family members with BDA1. **a** V-6, **b** V-2, **c** IV-4, **d** V-4, and **e** III-6 showing shortening of all digits, missing interphalangeal creases in the third to fifth digits, and mild clinodactyly of the fourth digits

**Fig. 4:**
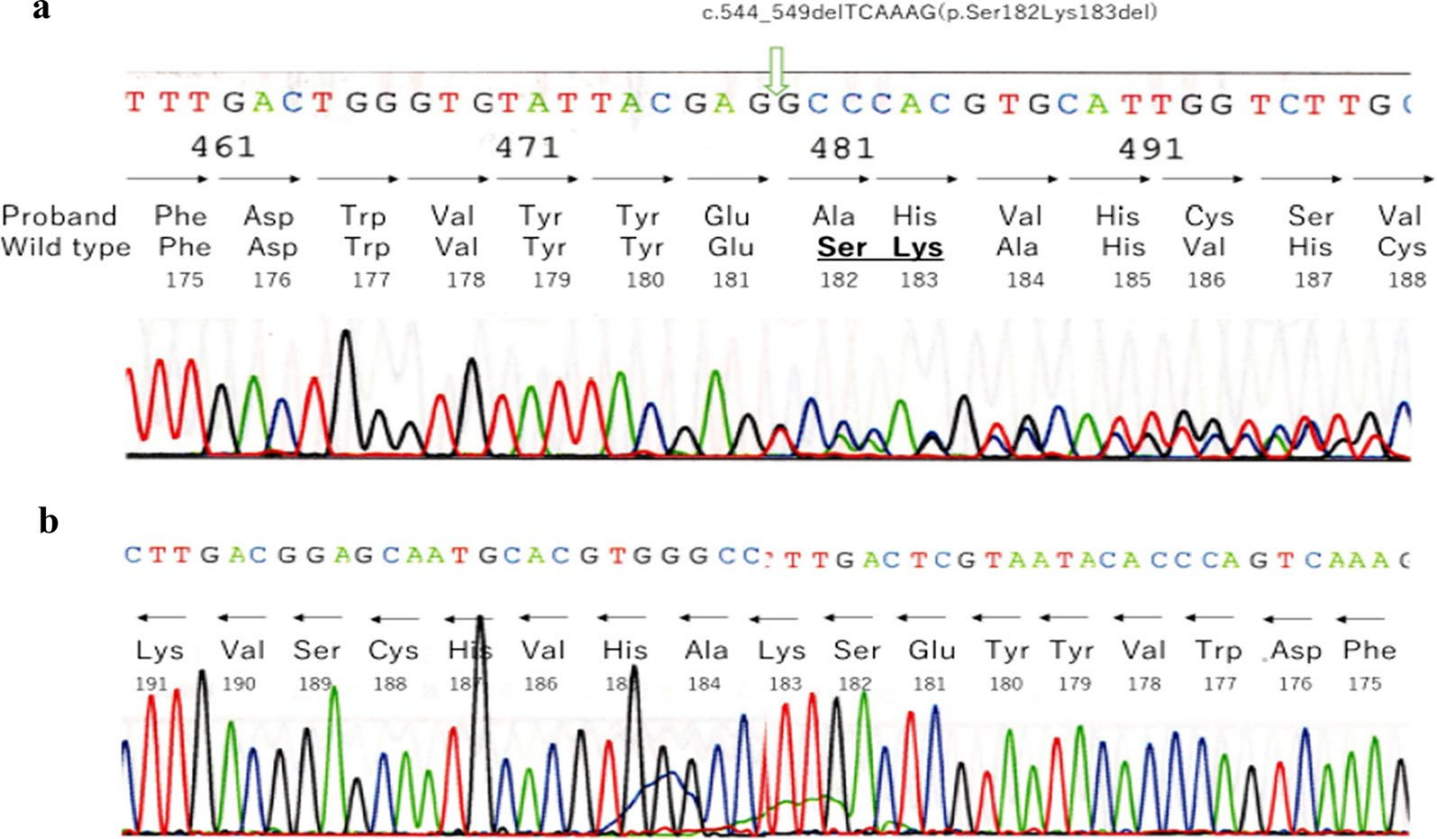
**a**, **b** Sanger sequencing chromatograph. **a** Proband V-5 showing the heterozygous deletion c.544_549delTCAAAG (p.Ser182Lys183del). **b** Non-affected mother IV-6 showing no deletion of Ser182 and Lys183 (in the reverse direction)

**Fig. 5:**
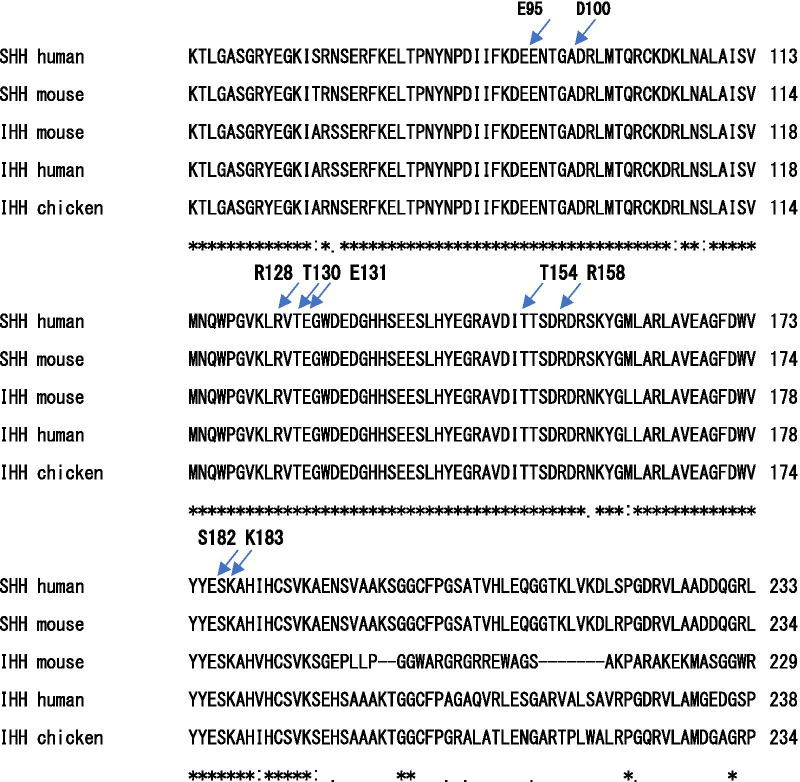
Comparison of the amino acid sequences of human, mouse and chicken Hedgehog proteins. The S812 and K183 residues of IHH are evolutionarily conserved, as are the other BDA1-associated amino acid residues

The proband (Fig. 1, V-5) is a 9-year-old boy who was born to non-consanguineous parents. He was referred to Kadono-Sanjo Children’s Clinic for a congenital hand malformation, with shortening of all digits of the hands and a short stature. His siblings (V-4, 6), cousin (V-2), aunt (IV-4), father (IV-5) and grandmother (III-6) also showed shortened digits and a short stature of variable severity. We diagnosed this family with BDA1 based on physical examinations, radiographic findings and family history. Written informed consent was obtained from all participants in compliance with the Ethics Review Board at Kyoto University (Approval No. G1228, Approval date Feb. 17, 2020). The pedigree is shown in Fig. 1.

### Genetic analysis

Genomic DNA was extracted from the peripheral blood of the proband (V-5), his siblings (V-4, 6), his parents (IV-5, 6), his aunt (IV-4) and his cousins (V-2, 3). All three exons and intron–exon boundaries of *IHH* were amplified by PCR and subjected to Sanger sequencing. For exon 1, different primers for PCR and sequencing were used due to difficulties in primer design, whereas for exons 2 and 3, the same forward primer was used for PCR and sequencing (Table 1).

**Table 1:** Primers for direct sequencing of *IHH*

Exon	Forward primer (5'-3')	Reverse primer (5'-3')
1	CGGCCTATTTATTGGCGG (PCR)	TGCCAGCCAGTCGAGAAAAT
	GCACCACGCCGCCCATGG (Sequencing)	
2	TTCCAGCTCCCTTGGGTGT	ATGTCCTCTTCCCCCGGAT
3	ATATGGTGACGGGGGCTCT	ATCTTCATGGCAGAGGAGATG

Three-dimensional structures and the spatial relationship between the variants in this case and the cluster regions of pathogenic variants reported previously were predicted using PyMOL (https://pymol.org/2/). Moreover, residue interactions across the interface between IHH and receptor proteins were predicted using PDBsum (https://www.uniprot.org/uniprot/Q14623).

## Results

### Clinical phenotype

There was no history of other medical conditions, including nystagmus, musculoskeletal abnormalities, a developmental delay or scoliosis. On examination, both hands of the proband showed shortening of all digits, and the distal interphalangeal creases in the third to fifth digits were hardly identifiable. Radial clinodactyly of the fourth digit was observed (Fig. 2a). There were no dysmorphic findings other than those in the hands. Radiographs of the proband’s hands revealed shortening of the middle phalanges of the second to fifth digits and fusion of the middle and terminal phalanges (Fig. 2c).

His siblings (V-4, 6), cousin (V-2), aunt (IV-4), father (IV-5) and grandmother (III-6) also showed shortened digits (Figs. 1, 2b and 3a–e). Radiographs of the father’s hand showed severe shortening of the middle phalanges of the second and third digits and fusion of the middle and terminal phalanges of the fourth and fifth digits (Fig. 2d). There were no abnormal findings in either the proximal phalanges or metacarpals of the proband or his father. The other four members of the five-generation family had similarly shortened digits (Fig. 1). This family pedigree is consistent with autosomal dominant inheritance. The proband was 116.3 cm in height (height standard deviation score [HSDS] -2.8) and had an arm span of 108.3 cm, with a mild, disproportionate short stature. His siblings (V-4,6), father (IV-5), aunt (IV-4), and cousin (V-2) had a short stature of variable severity, with HSDSs of − 1.7, − 1.8, − 2.2, − 3.0 and − 2.2, respectively.

### *Genetic analysis *via* Sanger sequencing*

Sequencing of *IHH* in individuals IV-4, 5, V-2, 4, 5 and 6 revealed a heterozygous variant, NM_002181.4:c.544_549delTCAAAG(p.Ser182Lys183del) [NC_000002.12:g.219057461_219057466del], a novel in-frame deletion that is not present in the population database (gnomAD (https://gnomad.broadinstitute.org/), the Exome Variant Server (http://evs.gs.washington.edu/EVS), dbSNP (https://www.ncbi.nlm.nih.gov/snp/), the 1000 Genomes Project (http://browser.1000genomes.org)), or the disease database ClinVar (http://www.ncbi.nlm.gov/clinvar). This variant was not identified in IV-6 nor V-3. No other coding variations were found in any of the sequenced regions (Fig. 4a, b). Alignment of the amino acid sequences of IHH and SHH, a homologue of IHH, with those of other species showed that Ser182 and Lys183 are highly conserved (Fig. 5).

### Three-dimensional modelling

**Fig. 6:**
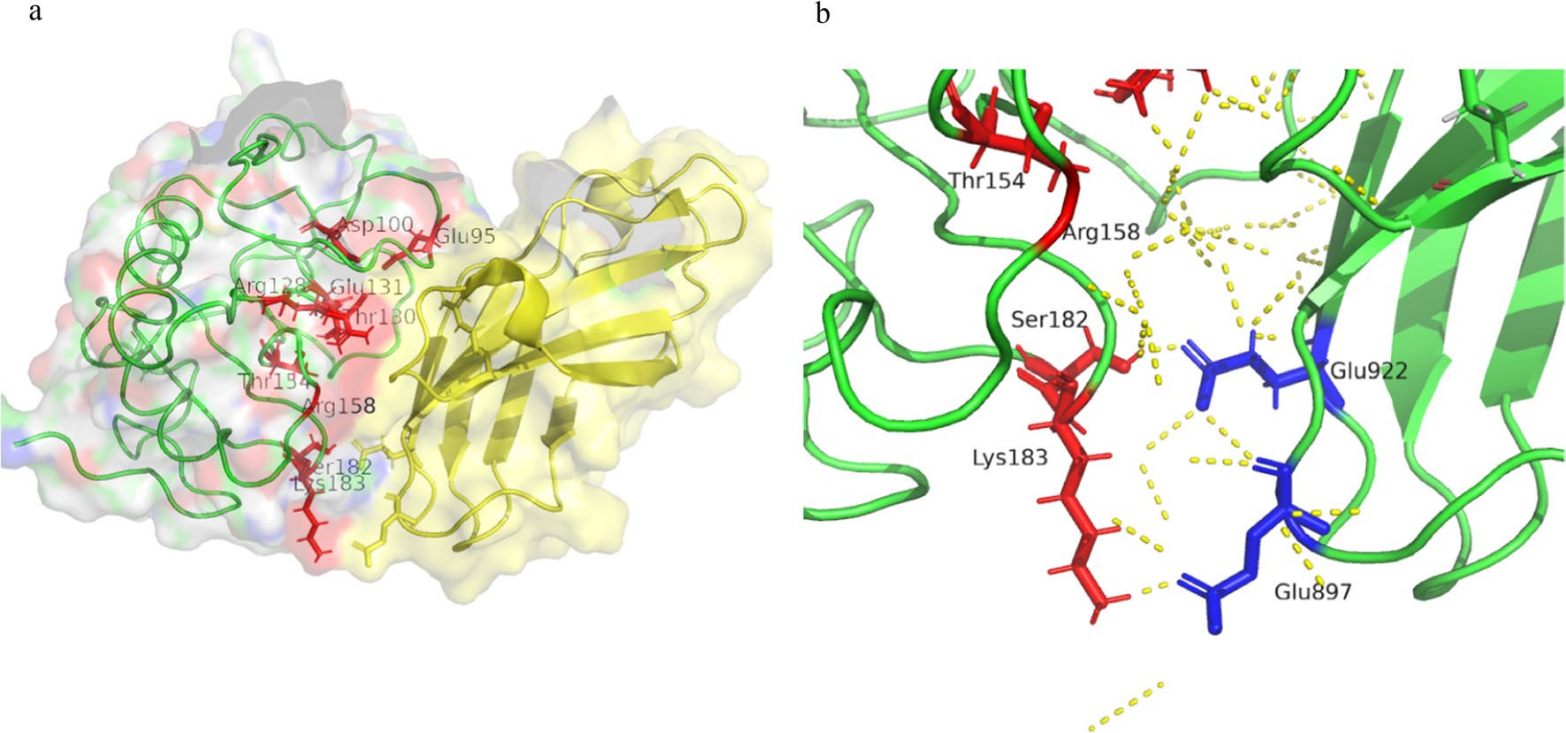
**a**, **b** Three-dimensional structure modelling of the N-terminal active fragment of the Indian Hedgehog protein using PyMOL. **a** shows the semitransparent molecular surface over a ribbon diagram of IHH-N (left) bound to CDO (right). The model shows that Ser182 and Lys183 are located on the same surface as other residues associated with BDA1. BDA1-associated residues are represented in red. **b** shows the IHH-N-CDO interface in the same orientation as in **a**. Ser182 and Lys183 on IHH-N interact with Glu922 and Glu897 on CDO respectively, via hydrogen bonds. Hydrogen bonds are depicted as dotted lines

Three-dimensional modelling using PyMOL showed that S182 and K183 are located on the same surface as Glu95, Asp100, Arg128, Thr130, Glu131, Thr154, and Arg158 which have been reported to be associated with BDA1 (Fig. 6a; https://pymol.org/2/, PDB ID 3N1F). In addition, PyMOL and PDBsum were used to analyze residue interactions across the interface between IHH and CDO, and S182 and K183 on IHH were predicted to interact with Glu922 and Glu897 on CDO via hydrogen bonds, with calculated inter-molecular distances of 2.47 Å and 2.87 Å, respectively (Fig. 6b. https://www.uniprot.org/uniprot/Q14623, PDBsum entry 3N1F, https://www.ebi.ac.uk/thorntonsrv/databases/cgi-bin/pdbsum/GetPage.pl?pdbcode=3N1F, https://pdbj.org/mine_molmilBU?pdbid=3n1f).

## Discussion

We report a novel heterozygous variant in the *IHH* gene in a Japanese family with BDA1. BDA1 is inherited in an autosomal dominant manner and is characterized by hypoplasia or aplasia of the middle phalanges of digits 2–5. Approximately half of the analysed BDA1 cases are due to mutations in *IHH* [1]. The Hedgehog (Hh) family of secreted proteins regulates various developmental processes, maintains adult tissue homeostasis, and functions as a morphogen gradient [15, 16].

Hh signalling is mediated by its N-terminal domain (HhN), and reception of the HhN signal is modulated by several cell surface proteins on responding cells, including Patched (Ptc), Smoothened (Smo), cell adhesion molecule-related, down- regulated by oncogenes (CDO), Hedgehog-interacting protein (Hip), and growth-arrest-specific 1 (Gas1) [2, 17–22]. In mammals, there are three homologues of the Hh family, Sonic (SHH), Indian (IHH), and Desert (DHH), each of which has tissue-specific functions. In limb development, SHH acts early, regulating patterning and growth [23]. Mutations in *SHH* are known to cause holoprosencephaly in humans [2, 24]. IHH, which is produced by prehypertrophic chondrocytes during endochondral ossification, acts later and is not believed to affect patterning but to regulate endochondral bone formation by controlling chondrogenic differentiation and proliferation [25–28].

IHH was shown to be a disease locus for BDA1, with *IHH* mutations c.G283A (p.E95K), c. C300A (p.D100E), and c.G391A (p.E131K) being initially identified in three Chinese BDA1 families [6]. Since the first report of these three variants, additional variants causing BDA1 have been identified. Reviewing published cases in addition to novel mutations identified by them, Byrnes et al. [3] concluded that all BDA1 variants involving codons 95, 100, 128, 130, 131 and 154 are limited to a 59-amino acid region of the N-terminal active fragment (IHH-N) that spans codons 95–154 Soon after this review, Stattin et al. [11] reported a Swedish family with a novel Arg158Cys mutation, showing that Byrnes et al.’s [3] proposal is not always the case. Since then, some novel BDA1 associated variants have been reported in various populations [7, 12, 13]. However, to date, variants other than those involving codons 95, 100, 128, 130, 131, 154, and 158 have never been identified. Using the X-ray crystal structure, McLellan et al. [4], showed that SHH-CDO interactions require calcium and that the CDO binding interface on SHH is conserved in nearly all Hh proteins. They further showed that this interface is a hotspot for mediating interactions between SHH and CDO, Ptc, Hip and Gas1, and mutations causing BDA1 including the one described by Sattin et al. map to this calcium-binding site and disrupt interactions with these partners. Kavran et al. [29] determined the crystal structures of IHH-N both alone and complexed with CDO, which allowed the direct visualization and interpretation of BDA1-causing mutations. They grouped BDA1-associated variants into two categories. The first group affects the calcium-binding region of IHH and includes E95K, E95G, D100N, D100E, and E131K. The second category of variants, with R128N, T154I, and T130N, interrupts hydrogen bond networks formed between IHH and CDO.

Here, we identified a novel in-frame deletion in *IHH*, designated NM_002181.4:c.544_549delTCAAAG(p.Ser182Lys183del) [NC_000002.12:g.219057461_219057466del, in a Japanese family with BDA1. This variant is distinct from previously identified variants in the following respects. First, codons 182 and 183 are somewhat distant from the limited 59-amino-acid region of *IHH* of pathogenic variants reported previously. Second, with the exceptions of a family with one amino acid insertion (p.Glu95_Asn96insLys) [13] and another with one amino acid deletion (p.Glu95del) [14], all the BDA1-causing *IHH* variants are missense mutations, and patients with two amino acid deletions have never been described. Although the variant we report here has such distinct features, we consider it to be pathogenic for the following reasons. First, this variant is absent from the population database. Second, this variant is in-frame two amino acid deletion that causes a protein length change in a well-conserved, non-repetitive region. Third, three-dimensional modelling showed that S182 and K183 are located on the same surface and bind receptor proteins as do other residues associated with BDA1 and that S182 and K183 on IHH interact with Glu922 and Glu897 on CDO via hydrogen bonding. This is consistent with the proposal of McLellan et al. [4] who reported that in the murine SHH-CDO complex, the murine SHH interface encompasses a region involving K88, E90, R124, H134, R154, R156, S178, and K179, with the S178 and K179 residues being equivalent to S182 and K183 in human IHH, respectively. Overall, we considered that residues K182 and S183 construct important elements of the interface that interacts with receptor proteins and that their deletion impairs the interactions between IHH and CDO, Ptc and Hip, thereby causing BDA1. As our study shows that S182 and K183 are involved in hydrogen bond networks, this would classify S182 and K183 into the latter group of variants proposed by Kavran et al. Our findings also predict the possibility of other BDA1-related variants being located on the interface between IHH and receptor proteins. According to the ACMG/AMP standards and guidelines for the interpretation of sequence variants, the novel variant is likely pathogenic (PM1, PM2, PM4, PP1, and PP4) [30].

Clinical phenotypes, with respect to digit malformation, showed no remarkable heterogeneity in the affected family members. On examination, all digits of the hands were short, and the distal interphalangeal creases in the third to fifth digits were barely identifiable. Radial clinodactyly of the fourth digit was observed in all affected individuals. Radiographically, the middle phalanges of all digits of the hands were present but uniformly shortened, especially in the second to fifth digits, and the middle phalanges were fused to terminal phalanges in the fifth digits. With the exception that proximal phalanges and metacarpals showed no remarkable findings, these phenotypes are consistent with the description characterized by Fitch and judged as mild [31]. A short stature of variable severity was observed among the Japanese family. Yang et al. [1] summarized several case reports of BDA1 and concluded that short stature was present only with *IHH* variants at Asp100. Affected siblings (V-4, 6) fell under the category of normal stature, although they were relatively short in height. However, given the severe short stature of the proband, his father, aunt, and his affected cousin, in contrast to the normal stature of unaffected cousins V-1 and 3 (HSDSs -1.0 and -0.6, respectively), the variant p.Ser182Lys183del could induce short stature with variable expressivity. This supports the notion that reduced IHH signalling may be responsible for reduced growth of long bones and result in short stature [6, 10, 32].

## Conclusions

We report a novel in-frame deletion of *IHH* NM_002181.4:c.544_549delTCAAAG (p.Ser182Lys183del) [NC_000002.12:g.219057461_219057466del] in a Japanese family with BDA1. Although the two deleted amino acid residues are located outside of the cluster region that is considered a hotspot of pathogenic variants, these two residues constitute the interface of interactions between IHH and CDO, Ptc and Hip, as do the other BDA1-associated residues. Future functional assays and additional experiments will be required to confirm the pathogenicity of this variant. Nevertheless, we are certain that our current findings will expand the genetic spectrum of BDA1.

## Data Availability

The datasets generated and/or analysed during the current study are available in the NCBI dbSNP repository, [https://www.ncbi.nlm.nih.gov/projects/SNP/snp_ss.cgi?subsnp_id=ss2137544202, local_identifier NC_000002.12:g.219057461_219057466delCTTTGA, NCBI_subsnp #2137544202, NM_002181.4: c.544_549delTCAAAG(p.Ser182Lys183del)]. The raw data of whole-exome sequencing of the patient in this study are not publicly available in order to protect participant confidentiality but are available from the corresponding author on reasonable request.

## Abbreviations

IHH: Indian hedgehog
BDA1: Brachydactyly type A1
IHH-N: N-terminal active fragment of IHH
HSDS: Height standard deviation score
Ptc: Patched
Smo: Smoothened
CDO: Cell adhesion molecule-related downregulated by oncogenes
Hip: Hedgehog-interacting protein
Gas1: Growth-arrest-specific 1
SHH: Sonic hedgehog
DHH: Desert hedgehog
